# Lipophilic fractions from the marine sponge *Halichondria sitiens* decrease secretion of pro-inflammatory cytokines by dendritic cells and decrease their ability to induce a Th1 type response by allogeneic CD4^+^ T cells

**DOI:** 10.1080/13880209.2017.1373832

**Published:** 2017-09-06

**Authors:** Xiaxia Di, Jon T. Oskarsson, Sesselja Omarsdottir, Jona Freysdottir, Ingibjorg Hardardottir

**Affiliations:** a Faculty of Pharmaceutical Sciences, University of Iceland, Reykjavik, Iceland;; b Department of Immunology, Landspitali – The National University Hospital of Iceland, Reykjavik, Iceland;; c Centre for Rheumatology Research, Landspitali – The National University Hospital of Iceland, Reykjavik, Iceland;; d Faculty of Medicine, Biomedical Center, University of Iceland, Reykjavik, Iceland

**Keywords:** Immunomodulation, anti-inflammatory effects, natural products

## Abstract

**Context:***Halichondria* (Halichondriidae) marine sponges contain components possessing various biological activities, but immunomodulation is not among the ones reported.

**Objective:** This study evaluated the immunomodulatory effects of fractions/compounds from *Halichondria sitiens* Schmidt.

**Materials and methods:** Crude dichloromethane/methanol extracts of *H. sitiens* were subjected to various chromatographic techniques to obtain fractions/compounds with immunomodulatory activity, using bioassay-guided isolation. The effects of the fractions/compounds were determined by measuring secretion of cytokines and expression of surface molecules by dendritic cells (DCs) and their ability to stimulate and modify cytokine secretion by allogeneic CD4^+^ T cells. The bioactive fractions were chemically analyzed to identify the immunomodulatory constituents by 1D, 2D NMR, and HRMS data.

**Results:** Several lipophilic fractions from *H. sitiens* at 10 μg/mL decreased secretion of the pro-inflammatory cytokines IL-12p40 and IL-6 by the DCs, with maximum inhibition being 64% and 25%, respectively. In addition, fractions B3b3F and B3b3J decreased the ability of DCs to induce T cell secretion of IFN-γ. Fraction B3b3 induced morphological changes in DCs, characterized by extreme elongation of dendrites and cell clustering. Chemical screening revealed the presence of glycerides and some minor unknown constituents in the biologically active fractions. One new glyceride, 2,3-dihydroxypropyl 2-methylhexadecanoate (**1**), was isolated from one fraction and two known compounds, 3-[(1-methoxyhexadecyl)oxy]propane-1,2-diol (**2**) and monoheptadecanoin (**3**), were identified in another, but none of them had immunomodulatory activity.

**Discussion and conclusions:** These results demonstrate that several lipophilic fractions from *H. sitiens* have anti-inflammatory effects on DCs and decrease their ability to induce a Th1 type immune response.

## Introduction

Over the last decades, there has been an increasing interest in investigating organisms of marine origin for potential drug leads (Tulp and Bohlin 2004; Cragg and Newman 2013). The ocean is the largest ecosystem on earth with immense biological diversity and it is considered an underutilized biological resource of new and diverse chemical entities with great potential for biomedical applications (Cragg and Newman 2013). Environmental conditions, such as temperature, salinity, pressure, light exposure, content of halogens and water quality, vary within the marine world and are markedly different from that of terrestrial environments. These environmental conditions have given marine organisms capacity to produce unique compounds with distinct metabolic and ecological roles.

Marine sponges are found to be a valuable source of bioactive metabolites that are important for their chemical defence against predators, space competitors and fouling (Mehbub et al. 2014). Bioactive extracts/compounds from marine sponges have been reported to have a wide range of activities, including anti-inflammatory, antioxidant, antiviral and antibacterial activities (Proksch et al. 2010; Joseph and Sujatha 2011).

Marine sponges of the genus *Halichondria* Fleming, 1828 (Halichondriidae) have been reported to contain various fatty acids (Imbs and Rodkina 2004), glycolipids (Nagle et al. 1992; Li et al. 1995), sterols (Jin et al. 2006), alkaloids (Tsubosaka et al. 2010) and terpenoids (Ishiyama et al. 2008). Some of these compounds possess antibacterial, cytotoxic, antifungal and antimicrobial properties (Jin et al. 2006). Although several recent studies have discovered anti-inflammatory activity of marine sponge-derived extracts (Costantini et al. 2015; Cheung et al. 2016), no studies have reported anti-inflammatory activities in extracts or fractions from *Halichondria (Eumastia) sitiens* (Schmidt, 1870), the sponge analyzed in the present study.

Inflammation is the response of the immune system against infection and tissue injury. Dendritic cells (DCs) play an important role in initiating a powerful adaptive immune response by activating naïve T cells (Banchereau and Steinman 1998). DCs further polarize the adaptive immune responses by their cytokine secretion, inducing appropriate T helper (Th) responses by directing the differentiation of naïve T cells into different Th effector cells, such as Th1, Th2 and Th17 (Kaiko et al. 2008). Th1 and Th17 cells are the main types of T cells participating in chronic immune responses; thus, the ability to dampen the responses of these cells may be beneficial to patients suffering from chronic inflammatory diseases.

In this study, the potential immunomodulatory activity of fractions/compounds from *H. sitiens* was explored by investigating their effects on maturation and activation of human monocyte-derived DCs and on the ability of the treated DCs to stimulate and differentiate allogeneic CD4^+^ T cells. Bioassay-guided isolation of the crude extract yielded 11 bioactive fractions with anti-inflammatory effects on DCs and selected fractions decreased the ability of the DCs to induce a Th1 type immune response. In addition, several of the biologically active fractions induced morphological changes in the DCs.

## Materials and methods

### Collection and identification of the marine sponge

*H. sitiens* was collected in July 2015 by scuba diving from the rocks of Kolbeinsey, north of Iceland, at a depth of approximately 10 m. The sponge was identified by Dr Hans Tore Rapp, University of Bergen. A voucher specimen is deposited at the Department of Natural Products Chemistry, Faculty of Pharmaceutical Sciences, University of Iceland.

### Extraction and bioassay-guided fractionation

The sponge was freeze-dried and then extracted twice with CH_2_Cl_2_:CH_3_OH (1:1) and evaporated to dryness on a rotary vacuum evaporator at 30 °C. The extract (105.3 g) was fractionated into five fractions using solvent partitioning method (a modified Kupchan method), starting with hexane (fraction A, 55.3 g), followed by chloroform (fraction B, 13.0 g) and dichloromethane (DCM) (fraction C, 0.4 g), *n*-butanol (fraction D, 3.4 g) and water (fraction E, 24.5 g). The B fraction (4.0 g) showed immunomodulatory activity and was subjected to SPE Silica gel CC (DCM-MeOH, 100:0→0:100) to obtain eight fractions (B1–B8). Fraction B3 (423.8 mg) was separated by Sephadex LH-20 CC (MeOH) into four fractions (B3a–B3d) and then B3b was separated by Sephadex LH-20 CC (MeOH–H_2_O, 90:10) to obtain four fractions (B3b1–B3b4). Fraction B3b3 (125.8 mg) was separated by preparative HPLC (MeOH–H_2_O, 10:90→0:100, 4.0 mL/min) with a 5 μm Agilent Eclipse XD8-C8 column (250 mm ×9.4 mm, Agilent Technologies, Inc., Santa Clara, CA) to obtain eighteen fractions (B3b3A–B3b3R). The following fractions with immunomodulatory activity were selected for further studies: B3b3F (1.5 mg, *t*_R_ = 18.92 min), B3b3G (2.0 mg, *t*_R_ = 19.66 min), B3b3H (1.2 mg, *t*_R_ = 20.71 min), B3b3I (1.5 mg, *t*_R_ = 23.63 min), B3b3J (1.4 mg, *t*_R_ = 24.27 min), B3b3K (3.4 mg, *t*_R_ = 25.21 min), B3b3L (2.7 mg, *t*_R_ = 27.08 min), B3b3M (3.8 mg, *t*_R_ = 27.25 min), B3b3N (2.2 mg, *t*_R_ = 28.05 min), B3b3O (1.2 mg, *t*_R_ = 29.10 min) and B3b3P (1.1 mg, *t*_R_ = 32.12 min). Fraction B3b3M was purified by HPLC (acetonitrile–H_2_O, 85:15, 1 mL/min) using YMC J'sphere ODS-H80 (250 mm ×4.6 mm, 8 nm, YMC Co., Ltd., Kyoto, Japan) and one pure compound was obtained, i.e., 2,3-dihydroxypropyl 2-methylhexadecanoate (**1**) (0.9 mg, *t*_R_ = 18.59 min).

The compound 2,3-dihydroxypropyl 2-methylhexadecanoate (**1**) is white powder; [*α*]^26 ^_D_ -10 (*c* 1.0, MeOH); IR *ν*_max_ cm^–1^: 3442, 2921, 2851, 1728, 1628, 1467, 1384, 1111, 672; for ^1^H and ^13^C NMR data, see Table 1; HRESIMS *m/z* 367.2811 [M + Na]^+^ (C_20_H_40_O_4_Na, 367.2819).

**Table 1.** ^1^H (400 MHz) and ^13^C (100 MHz) NMR spectroscopic data of compound **1** in CDCl_3_.

2,3-Dihydroxypropyl 2-methylhexadecanoate (**1**)		
Carbon number	*δ*_H_ (*J* in Hz)	*δ*_C_
1	3.70, dd (11.5, 4.5) 3.60, dd (11.5, 6.0)	63.3
2	3.93, quint (4.5)	70.3
3	4.22, dd (12.0, 4.5) 4.16, dd (12.0, 6.0)	65.1
4		177.5
5	2.48, sextet (6.0)	39.5
6	1.16, d (7.0)	17.1
7	1.66, m	33.8
8–18	1.24–1.41, m	27.2–31.9
19	1.30, m	22.7
20	0.87, t (6.5)	14.1

### Chemical analysis of the active fractions

The NMR spectra were run on a Bruker AM-400 spectrometer (Bruker Corporation, Billerica, MA) with TMS as internal standard in deuterated chloroform as a solvent, unless otherwise stated, at 400.13 and 100.61 MHz for ^1^H and ^13^C, respectively. Chemical shifts were reported with reference to the respective residual solvent peaks (*δ*_H_ 7.27 and *δ*_C_ 77.0 for CDCl_3_). High-resolution mass spectra (HRMS) were carried out on micrOTOF-Q mass spectrometer from Bruker Daltonics (Billerica, MA).

### Maturation and activation of DCs

Peripheral blood mononuclear cells (PBMCs) were obtained by density-gradient centrifugation of blood from healthy human donors using Ficoll Histopaque (Sigma-Aldrich, St. Louis, MO). CD14^+^ monocytes were isolated using CD14 Microbeads (Miltenyi Biotec, Bergisch Gladbach, Germany). Their purity was determined by flow cytometry to be >95%. DCs were obtained by culturing CD14^+^ monocytes in 48-well culture plates (Nunc) at a concentration of 5 × 10^5^ cells/mL in RPMI medium (Gibco^®^, Invitrogen, Carlsbad, CA) in the presence of 12.5 ng/mL of IL-4 and 25 ng/mL of GM-CSF (both from R&D Systems, Minneapolis, MN) for 7 d, with fresh medium and cytokines added at day 3. The DCs were matured and activated by culturing them in 48-well culture plates at 2.5 × 10^5^ cells/mL for 24 h with IL-1β at 10 ng/mL, TNF-α at 50 ng/mL (both from R&D Systems, Minneapolis, MN) and lipopolysaccharide (LPS) at 500 ng/mL (Sigma-Aldrich, St. Louis, MO). Fractions/compounds from *H. sitiens* were dissolved in dimethyl sulphoxide (DMSO) and then diluted in medium and added to the DCs at the same time as the cytokines and LPS. DCs were also cultured in the presence of the same concentration of DMSO as in the cultures with the *H. sitiens* fractions/compounds (0.005%; solvent control). After 24 h, the mature and activated DCs were harvested and the effects of the fractions/compounds on DC maturation and activation were determined by measuring cytokine concentration in the culture medium by ELISA and the expression of surface molecules by flow cytometry.

### Viability assessment and morphology of DCs

The viability of DCs cultured with or without fractions from *H. sitiens* was assessed by Trypan blue staining. DCs were matured and activated in the presence/absence of fractions at the concentration of 10 μg/mL. Untreated and solvent (DMSO) treated DCs served as controls. Morphology of the DCs was evaluated in 10 × magnification using Leica microscope.

### Co-culture of DCs and allogeneic CD4^+^ T cells

CD4^+^ T cells were obtained from PBMCs using CD4 Microbeads (Miltenyi Biotec, Bergisch Gladbach, Germany), following the same procedure as for the isolation of CD14^+^ monocytes described above. The purity of the CD4^+^ T cells was determined by flow cytometry to be >95%. DCs that had been matured and activated with TNF-α, IL-1β and LPS in the presence or absence of fractions at 10 µg/mL were co-cultured at 2 × 10^5^ cell/mL with allogeneic CD4^+^ T cells at 2 × 10^6^ cells/mL in 96-well round bottom culture plates for 6 d. The effects of the fractions on the ability of the DCs to activate the CD4^+^ T cells were determined by measuring cytokine concentrations in the co-cultures by ELISA and expression of activation molecules by flow cytometry.

### Determination of cytokine concentration by ELISA

The concentrations of IL-12p40, IL-6 and IL-10 in culture supernatants from DCs and of IFN-γ, IL-17 and IL-10 in supernatants from co-cultured DCs and allogeneic CD4^+^ T cells were measured by sandwich ELISA using DuoSets from R&D Systems according to the protocol from the manufacturer. The results are expressed as secretion index (SI), i.e., the cytokine concentration in supernatants from DCs cultured with *H. sitiens* fractions or co-cultures of these DCs with allogeneic CD4^+^ T cells divided by the cytokine concentration in supernatants of DCs cultured without *H. sitiens* fractions or co-cultures of these DCs with allogeneic CD4^+^ T cells. Using SI minimizes the effect of the variance in cytokine secretion by DCs from different individuals.

### Determination of surface molecule expression by flow cytometry

Surface molecule expression of monocytes, DCs and CD4^+^ T cells was determined by flow cytometry. Five ×10^5^ cells were stained with fluorochrome-labelled antibodies against CD14 (freshly isolated monocytes or differentiated DCs), CD4 (freshly isolated or activated and differentiated T cells), CD86 and HLA-DR (matured and activated DCs) and CD54, CD49d and CD69 (activated and differentiated T cells) and analyzed by FACScalibur (BD Bioscience, Franklin Lakes, NJ). The results are expressed as % of positive cells as compared with cells stained with isotype control antibodies and mean fluorescence intensity.

### Statistical analysis

Data are presented as the mean values + standard error of the mean (SEM). As the data were not normally distributed, Mann–Whitney *U* test was used to determine statistical differences between treatments (SigmaStat 3.1, Systat Software, San Jose, CA) and differences between means considered statistically significant if *p* < 0.05.

## Results

### Chemical analysis of fractions and structure elucidation of the main constituents

Chemical analysis of fractions B3b3F-B3b3P from *H. sitiens* was conducted using NMR and HR-ESI-MS. Chemical analysis of four of them, i.e., fractions B3b3F, B3b3J, B3b3P and B3b3M, is reported. These fractions were chosen because of their potent bioactivity (B3b3F and B3b3J), and/or because they contained major components as revealed by ^1^H NMR and mass spectra (B3b3J and B3b3M), or in the case of B3b3P because it had much less bioactivity than the other fractions. The small amount of the fractions and difficulties with purification prevented further isolation of pure compounds (except compound **1** reported below). Therefore, all spectra were recorded for the fractions and the structure of the major constituents determined.

**Figure 1.**
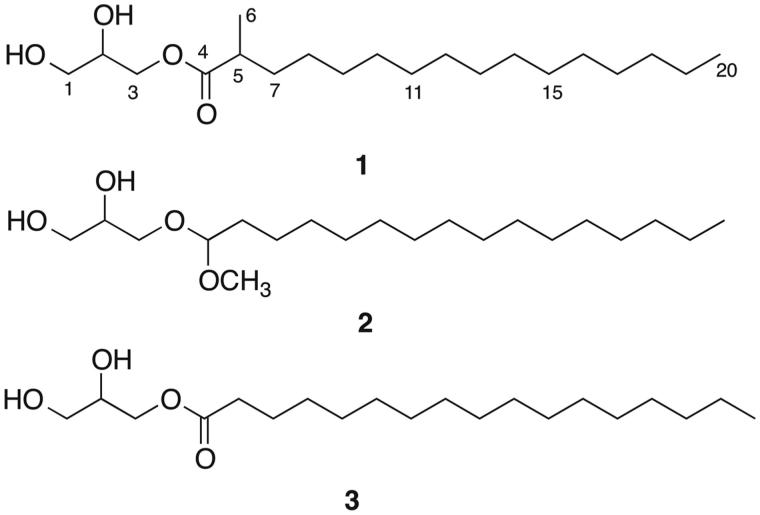
Chemical structures of the compounds isolated or identified from *H. sitiens* fractions B3b3M (**1**) and B3b3J (**2** and **3**).

HRESIMS of fraction B3b3J gave two of [M + Na]^+^ pseudomolecular ion peaks at *m*/*z* 369.2965 and 367.2811, suggesting a mixture of homologous compounds consistent with the molecular formulae C_20_H_42_O_4_ and C_20_H_40_O_4_, respectively. Combined analysis of the 1D and 2D NMR showed that fraction B3b3J contained a previously described glycerol ester, i.e., 3-[(1-methoxyhexadecyl)oxy]propane-1,2-diol (**2**) (Magnusson and Haraldsson 2010), and a known glycerol ether, i.e., monoheptadecanoin (**3**) (Qi et al. 2010) (Figure 1) in the ratio of 1:2 based on the signal heights in the ^1^H NMR spectrum. These two known compounds were identified by comparison with the published physical and spectral data. B3b3J also contained a small portion of unknown compounds.

Analysis of the ^1^H NMR data of fraction B3b3F revealed that the main compounds in it were glycerides, similar to the compounds detected in fraction B3b3J but with double bonds in the fatty acid chains. It also contained a small portion of unknown compounds. Because fraction B3b3F contained more than three compounds, the structure could not be determined from ^1^H NMR and HRESIMS.

From the ^1^H NMR spectrum for fraction B3b3P, it was determined that it contained glycerides similar to the monoheptadecanoin (**3**). Because the mass spectra showed a complex mixture, the exact chain length of the fatty acids could not be confirmed. Fraction B3b3P also contained minor compounds that could not be identified because of the limited amount available.

**Figure 2.**
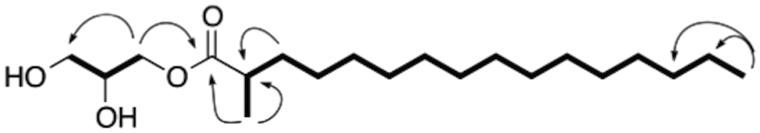
Key HMBC (H→C) and ^1^H-^1^H COSY (—) correlations of compound **1**.

A new glyceride, 2,3-dihydroxypropyl 2-methylhexadecanoate (**1**), was detected and purified from fraction B3b3M. Compound **1** was isolated as a white amorphous solid. Its molecular formula was established on the basis of MS and NMR spectral analysis. The HR-ESI-MS showed the [M + Na]^+^ peak at 367.2811, which matched well with the expected molecular formula C_20_H_40_O_4_Na (Δ + 1.3 ppm). The 1D and 2D NMR spectra (Table 1) showed the presence of two terminal methyls, numerous methylenes, two of which were oxygenated, two methines, one of which was oxygenated, and one ester group. The ^1^H–^1^H COSY spectrum (Figure 2) revealed two partial structures: the first part was suggested to be one 1-substituted glyceryl group (C1–C3), which is also supported by the HMBC correlations (Figure 2); the second unit was assigned as 2-methylhexadecanoyl group (C4–C20), because of the remaining signal of the two terminal methyls, one methine, and 13 methylenes. The presence of 2-methylhexadecanoyl group was identified by the HMBC correlations from Me-6 to C-4, C-5 and C-7. The connection of these two parts was deduced by the HMBC H-3/C-4 correlation (Figure 2). The above data revealed that compound **1** was a monoglyceride (Figure 1). Thus, the structure was determined to be 2,3-dihydroxypropyl 2-methylhexadecanoate.

### Effects of fractions and pure compounds on maturation and activation of DCs

There was no effect of any of the fractions B3b3F–B3b3P on DC cell viability when compared with the viability of DCs cultured without fractions or with solvent control (data not shown).

**Figure 3.**
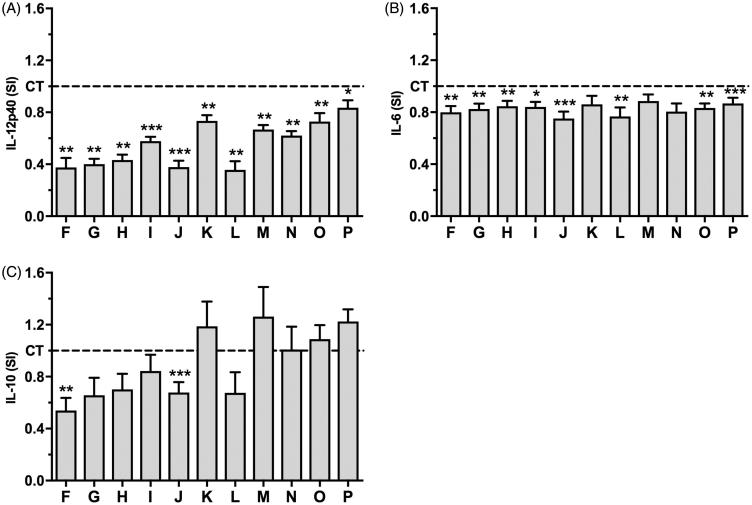
The effects of *H. sitiens* fractions on DC secretion of IL-12p40, IL-6 and IL-10. DCs were matured and activated by TNF-α, IL-1β and LPS in the absence (solvent control (CT)) or presence of fractions B3b3F–B3b3P (F–P) for 24 h. The supernatants were collected and the concentrations of IL-12p40 (A), IL-6 (B) and IL-10 (C) were determined by ELISA. The data are presented as SI, i.e. the concentration of each cytokine in the supernatant of cells matured and activated in the presence of fractions divided by the concentration of the cytokine in the supernatant of cells matured and activated in the absence of fractions. The results are shown as mean + SEM, *n* = 6–8. Different from CT: *p < 0.05, **p < 0.01, ***p < 0.001.

All the fractions decreased secretion of the pro-inflammatory cytokine IL-12p40 (Figure 3(A)) with the inhibition ranging from 16% to 64% and all fractions, except B3b3K, B3b3M and B3b3N, decreased DC secretion of the pro-inflammatory cytokine IL-6 with the inhibition ranging from 11% to 25% (Figure 3(B)). The effects of the fractions on DC secretion of IL-12p40 were more pronounced than their effects on DC secretion of IL-6. B3b3F and B3b3J were the only two fractions that affected DC secretion of the anti-inflammatory cytokine IL-10, both decreasing secretion of IL-10 (Figure 3(C)).

The pure compounds 3-[(1-methoxyhexadecyl)oxy]propane-1,2-diol (**2**) (a gift from professor Gudmundur G. Haraldsson at the Science Institute, University of Iceland), monoheptadecanoin (**3**) (Nu-Chek Prep Inc., Waterville, MN), and the one isolated from B3b3M, 2,3-dihydroxypropyl 2-methylhexadecanoate (**1**), did not affect DC secretion of any of the cytokines examined (data not shown).

To determine whether decreased cytokine secretion by DCs matured and activated in the presence of fractions B3b3F–B3b3P was because of unsuccessful maturation and activation of the DCs, their expression of CD86 and HLA-DR was analyzed. Maturing and activating the DCs in the presence of fractions B3b3F-B3b3P did not affect DC expression of these molecules (data not shown).

### The effects of DCs matured in the presence of fractions on stimulation and differentiation of allogeneic CD4^+^ T cells

**Figure 4.**
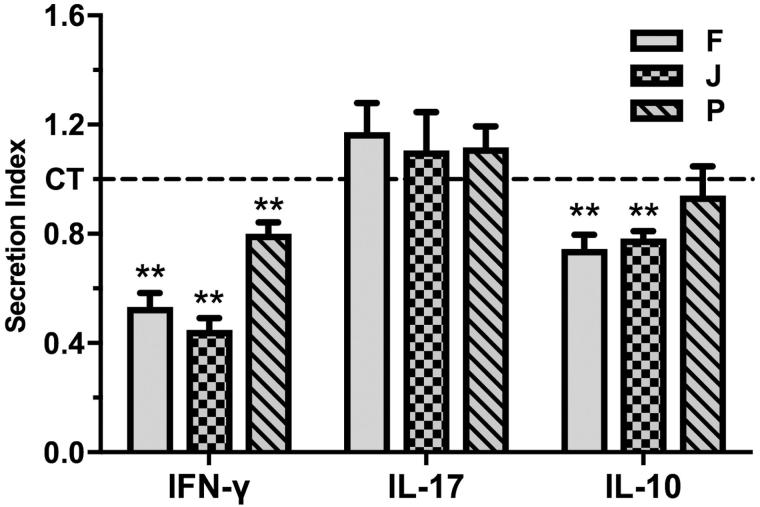
The effects of *H. sitiens* fractions on the ability of DCs to induce cytokine secretion by allogeneic CD4^+^ T cells. DCs matured and activated in the absence (solvent control (CT)) or presence of fractions B3b3F (F), B3b3J (J) and B3b3P (P) at a concentration of 10 µg/mL for 24 h were co-cultured with isolated allogeneic CD4^+^ T cells for 6 d and the concentration of IFN-γ, IL-17 and IL-10 in the supernatants determined by ELISA. The data are presented as SI, i.e. the concentration of each cytokine in the supernatant of cells treated with fractions divided by the concentration of each cytokine in the supernatant of cells treated with solvent control. The results are shown as mean + SEM, *n* = 6. Different from CT: **p < 0.01.

Next it was investigated whether, in addition to decreasing DC secretion of cytokines, the fractions also affected the ability of the DCs to activate and differentiate CD4^+^ T cells. Three fractions were chosen for this analysis, one that effectively decreased both IL-12p40 and IL-10 secretion by the DCs (B3b3F), one that effectively decreased DC secretion of IL-12p40 but had less effect on IL-10 (B3b3J) and one was chosen for comparison as it had little effect on DC secretion of IL-12p40 and no effect on IL-10 (B3b3P). DCs matured and activated in the presence of all three fractions decreased T cell secretion of IFN-γ, although fractions B3b3F and B3b3J had more pronounced effect than fraction B3b3P (Figure 4). The concentration of IL-10 was also decreased in co-cultures of DCs matured and activated in the presence of fractions B3b3F and B3b3J and allogeneic CD4^+^ T cells, but as IL-10 is produced by both DCs and T cells the cellular source of the IL-10 could not be determined (Figure 4). Maturation and activation of DCs in the presence of fractions B3b3F, B3b3J and B3b3P did not affect the ability of the DCs to induce T cell secretion of IL-17 (Figure 4).

To determine whether decreased IFN-γ secretion by T cells co-cultured with DCs matured and activated in the presence of fractions B3b3F or B3b3J was the result of unsuccessful activation of the allogeneic T cells, the expression of the adhesion molecules CD54 and CD49d (α-chain of VLA-4) and the early activation marker CD69 on T cells was analyzed. The expression of these surface molecules was not affected by co-culture of the T cells with DCs matured and activated in the presence of fractions B3b3F or B3b3FJ (data not shown).

### Effects of fractions on DC morphology

**Figure 5.**
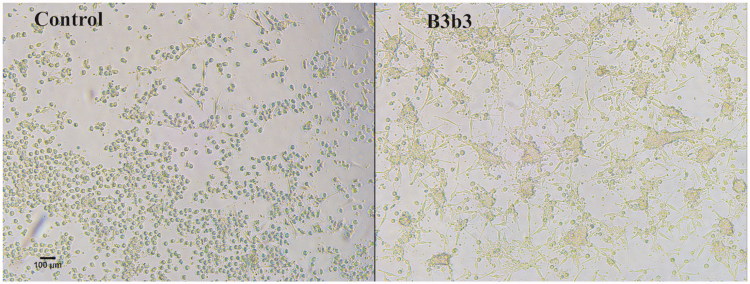
The effect of *H. sitiens* fraction B3b3 on DC morphology. DCs were matured and activated in the presence of fraction B3b3 at a concentration of 10 µg/mL or solvent control (control) for 2 h and viewed in light microscope. 10× magnification. Scale bar 100 µm.

While screening for anti-inflammatory effects of fractions on DCs, it was noticed that some of the fractions affected their morphology. These morphological changes, characterized by extreme elongation of 1–3 dendrites, were particularly apparent for DCs matured and activated in the presence of the mother fraction B3b3 (Figure 5). B3b3 also induced more clustering of the DCs and at an earlier time-point after maturation and activation than was observed for DCs matured and activated in the absence of the fraction (Figure 5). Some of the B3b3 sub-fractions had similar, although less pronounced, effects on the morphology of the DCs (data not shown).

## Discussion

In this study, we show that several lipophilic fractions from the sponge *H. sitiens* contain compounds that decrease secretion of the pro-inflammatory cytokines IL-12p40 and IL-6 by matured and activated DCs. Only two of the 11 fractions studied decreased DC secretion of the anti-inflammatory cytokine IL-10 and to a much less extent than that of IL-12p40. The overall effects of the fractions are, therefore, considered as anti-inflammatory. Further examination of the immunomodulatory effects of three of the fractions revealed that DCs matured and activated in the presence of fractions B3b3F and B3b3J reduced IFN-γ secretion by co-cultured allogeneic CD4^+^ T cells by around 50%, with only a modest reduction in IL-10 secretion, thus decreasing the ability of the DCs to induce a Th1-type immune response.

The fractions investigated in this study are derived from the chloroform fraction (B) of the modified Kupchan method, containing mostly glycerol esters and glycerol ethers along with long chain saturated and unsaturated fatty acids. Most of the fractions had a number of chemical constituents and no major ones, as demonstrated by the number of peaks on the HPLC chromatograms and mass spectra. As the amount of the material available was limited it was not feasible to subject the fractions to further isolation. The only fraction that contained one major peak was B3b3M. The compound isolated from fraction B3b3M is a new glyceride, 2,3-dihydroxypropyl 2-methylhexadecanoate (**1**), but it did not have an immunomodulatory effect on the DCs. Neither did the two main compounds identified in B3b3J, 3-[(1-methoxyhexadecyl)oxy]propane-1,2-diol (**2**) and monoheptadecanoin (**3**). The finding that compounds **2** and **3** did not have immunomodulatory effects was surprising as they were the main constituents of the fraction (B3b3J) that had the most immunomodulatory effects of all the fractions examined. Adding the two compounds in different ratios to the DCs did not affect the cytokine secretion of the DCs. Other constituents in fraction B3b3J, although in minor quantities, are, therefore, likely to be the ones exerting the immunomodulatory activity.

Long-chain glycerol esters have been found in marine sponges, either in the phospholipid fraction or as free derivatives, and glycerol ethers with different lengths of chains are present in great amount in shark liver oil (Hallgren and Stallberg 1974). These have attracted attention of medicinal chemists because of their structure (Mattson and Volpenhein 1962; Magnusson and Haraldsson 2010) and potent pharmacological activities, including anti-inflammatory activity (Chang et al. 2008; Morin et al. 2011, 2015).

DCs play a crucial role in linking the innate and adaptive immune systems and polarizing the differentiation of naïve T cells into appropriate effector phenotypes. DCs are, therefore, prime targets when evaluating the immunomodulatory effects of natural compounds. DC secretion of the pro-inflammatory cytokine IL-12 is considered a major contributor to the polarization of naïve T cells into Th1 effector cells, a phenotype characterized by secretion of the pro-inflammatory cytokine IFN-γ (Manetti et al. 1994; Macatonia et al. 1995; Gee et al. 2009). The decreased IL-12p40 secretion by DCs matured in the presence of fractions B3b3F and B3b3J in the present study was, therefore, expected to lead to a decrease in their ability to polarize CD4^+^ T cells into Th1 effector phenotype, as was evident by the decreased IFN-γ secretion by the CD4^+^ T cells after co-culture with allogeneic DCs matured and activated in the presence of fractions B3b3F and B3b3J. Long-lasting Th1 type immune responses play a role in the pathogenesis of many inflammatory diseases, such as Crohn’s disease, type 1 diabetes and rheumatoid arthritis (Zundler and Neurath 2015). Therefore, fractions from *H. sitiens* that reduce the Th1-type immune response may contain compounds that could be evaluated for their effectiveness in such diseases.

In addition to decreasing IL-12p40 secretion by the DCs, the majority of the fractions examined also decreased DC secretion of IL-6. IL-6 along with IL-1β is important for differentiation of naïve T cells into Th17 phenotype, characterized by secretion of IL-17 (Acosta-Rodriguez et al. 2007). However, although fractions B3b3F, B3b3J and B3b3P reduced DC secretion of IL-6, none of them affected the concentration of IL-17 in the co-cultures of DCs matured in the presence of the fractions and CD4^+^ T cells. As the decrease in IL-6 secretion by DCs matured in the presence of these fractions was moderate (less than 20%), these results were not surprising. In addition, others have shown that decreased IL-6 secretion by DCs cultured in the presence of the local anaesthetic and cardiac depressant lidocaine did not affect differentiation of T cells into Th17 cells (Jeon et al. 2015). Interestingly, as for the *H. sitiens* fractions B3b3F, B3b3J and B3b3P in the present study, lidocaine decreased IL-12 secretion by DCs and inhibited their ability to induce differentiation of CD4^+^ T cells into a Th1 phenotype (Jeon et al. 2015).

The fractions B3b3F and B3b3J reduced DC secretion of the anti-inflammatory cytokine IL-10 as well as the concentration of IL-10 in the co-culture of DCs and T cells. IL-10 is well established as an anti-inflammatory cytokine, exerting multiple effects in down-regulation and resolution of inflammatory responses and is a major determinant in differentiation of naïve T cells into a T regulatory phenotype (Iyer and Cheng 2012). Although reduction in IL-10 secretion could point towards pro-inflammatory responses, we have observed in previous studies that decreased IL-10 secretion along with a reduction in IL-12p40 secretion did not hinder the IL-12p40 in reducing IFN-γ secretion by co-cultured CD4^+^ T cells (Freysdottir et al. 2011; Kale et al. 2013) as was the case in the present study.

The morphological changes of the DCs (elongated dendrites on the cells) were observed after treatment with fraction B3b3 and some of the B3b3 sub-fractions, but these changes did not correlate with the immunomodulatory activity of the fractions nor with their maturation and activation as determined by the expression of HLA-DR and CD86. The biological significance of these changes is unknown.

## Conclusions

Using bioassay-guided fractionation, several lipophilic fractions with potent anti-inflammatory effects, i.e., reducing the capacity of the DCs to secrete IL-12p40 and subsequently to induce Th1-like response by T cells, were obtained from the sponge *H. sitiens*. Although pure compounds were not isolated from these fractions, it was determined that the majority of the compounds in these fractions were of glyceride nature. These results highlight the potential use of lipophilic components from *H. sitiens* for immunomodulatory effects.
